# MDM4 Overexpressed in Acute Myeloid Leukemia Patients with Complex Karyotype and Wild-Type *TP53*

**DOI:** 10.1371/journal.pone.0113088

**Published:** 2014-11-18

**Authors:** Li Li, Yanhong Tan, Xiuhua Chen, Zhifang Xu, Siyao Yang, Fanggang Ren, Haixiu Guo, Xiaojuan Wang, Yi Chen, Guoxia Li, Hongwei Wang

**Affiliations:** 1 Department of Hematology, the Second Hospital of Shanxi Medical University, Taiyuan, Shanxi, P.R. China; 2 Department of biology, School of Basic Medicine, Shanxi Medical University, Taiyuan, Shanxi, P.R. China; Queen's University Belfast, United Kingdom

## Abstract

Acute myeloid leukemia patients with complex karyotype (CK-AML) account for approximately 10–15% of adult AML cases, and are often associated with a poor prognosis. Except for about 70% of CK-AML patients with biallelic inactivation of *TP53*, the leukemogenic mechanism in the nearly 30% of CK-AML patients with wild-type *TP53* has remained elusive. In this study, 15 cases with complex karyotype and wild-type *TP53* were screened out of 140 *de novo* AML patients and the expression levels of MDM4, a main negative regulator of p53-signaling pathway, were detected. We ruled out mutations in genes associated with a poor prognosis of CK-AML, including *RUNX1* or *FLT3-ITD*. The mRNA expression levels of the full-length of *MDM4* (*MDM4FL*) and short isoform *MDM4* (*MDM4S*) were elevated in CK-AML relative to normal karyotype AML (NK-AML) patients. We also explored the impact of MDM4 overexpression on the cell cycle, cell proliferation and the spindle checkpoint of HepG2 cells, which is a human cancer cell line with normal MDM4 and TP53 expression. The mitotic index and the expression of p21, BubR1 and Securin were all reduced following Nocodazole treatment. Moreover, karyotype analysis showed that MDM4 overexpression might lead to aneuploidy or polyploidy. These results suggest that MDM4 overexpression is related to CK-AML with wild-type *TP53* and might play a pathogenic role by inhibiting p53-signal pathway.

## Introduction

Acute myeloid leukemia patients with complex karyotype (CK-AML) account for approximately 10–15% of adult AML, and the incidence increases with age. CK-AML is characterized by chemoresistance, higher rates of refractory disease, and poor prognosis [1]–[3]. However, the molecular mechanisms mediating of leukemogenesis in CK-AML patients have remained elusive. A series of large sample studies show that nearly 70% of CK-AML cases carry *TP53* mutations and have biallelic inactivation of *TP53* [4], [5]. p53 plays an important role in spindle damage induced mitotic arrest in proliferating T cells [6] and p53 lost myeloid progenitors exhibit aberrant self-renewal, thereby promoting AML[7]. Yet the question remains as to the leukemogenic mechanisms of the nearly 30% of CK-AML patients without *TP53* alterations.

MDM4 is a negative regulator of p53, and by binding p53, close the transcriptional activity domain and thereby inhibits p53 function [8]. The short isoform of MDM4 (MDM4S) is one of the MDM4 alternative splicing isoforms that results from the exclusion of exon 6 and termination of translation in exon 7. MDM4S is essentially a truncated protein that mainly consists of the p53-binding domain. MDM4S has been reported to bind and inhibit p53 more efficiently than full-length MDM4 (MDM4FL) [9]. Several recent studies suggest that an increased MDM4S/MDM4FL ratio may serve as both a more effective biomarker for p53 pathway attenuation in cancers than p53 gene mutation and as a poor prognostic indicator. [10], [11]. The molecular mechanisms of myeloproliferative neoplasm (MPN) converting into AML were examined in 330 cases [12]. Among the 22 patients with transferred to AML, 10 (45.5%) cases had evidence of a p53-related defect mediated by gains (amplification) of chromosome 1q (which contains the potent p53 inhibitor MDM4) or *TP53* gene mutations. These reports suggest that overexpression MDM4 may be involved in the leukemogenic mechanisms of CK-AML patients without *TP53* alterations. This question has not been fully explored to date.

In this study, we detected the expression levels of *MDM4S* and *MDM4FL* in CK-AML patients with wild-type *TP53*. We also measured cell proliferation, cell cycle, proteins related to p53 pathway and spindle checkpoint expression levels, and analyzed karyotypes in MDM4-overexpressing tumor cell line with wild-type TP53. We used these approaches to investigate the possible pathogenesis of MDM4-overexpression in CK-AML patients lacking TP53 mutations.

## Materials and Methods

### Ethics Statement

This study complies with the Declaration of Helsinki, and has been approved by the Ethics Committee of Shanxi Medical University. The written informed consent was obtained from all patients and from the legal guardians in the case of minors.

### Patients

Bone marrow samples were collected at the time of diagnosis of 140 non-M3 *de novo* AML patients. The fusion genes *RUNX11/RUNX1T1*, *PML/RARα* or *CBFβ/MYH11* of the patients were identified to be negative at the time of enrollment.

### Karyotype analysis

Conventional cytogenetics was performed at the time of diagnosis in 140 patients. Bone marrow cells were cultured in RPMI 1640 medium with 10% fetal bovine serum and penicillin-streptomycin for 24 hours, followed by treatment with 0.01 mg/ml colcemid for 60 min. Cells were harvested and placed in 0.075 M KCl for 15 min. After several changes in methanol-acetic acid fixative, slides were prepared by hot-plate drying. Metaphase chromosomes were banded by the trypsin-Giemsa or Phosphate R technique, and karyotyped according to the International System of Human Cytogenetic Nomenclature (ISCN 2005).

### PCR and Gene sequencing

Exons 3–9 of the *TP53* gene and exon 3–9 of *RUNX1* were amplified by PCR from genomic DNA and sequenced directly in all cases with complex karyotype. *TP53* deletions were detected by interphase FISH in complex karyotype cases. Fms-related tyrosine kinase 3 length mutation (*FLT3-ITD*) analysis was performed as published [13] in CK-AML with wild-type *TP53* and NK-AML patients.

### Real-time RT-PCR

For quantitative RT-PCR, cDNA was prepared using PrimeScript 1^st^ Strand cDNA Synthesis Kit (TaKaRa, Shiga, Japan) and used in quantitative real-time PCR reactions with SYBR Premix Ex Taq (TaKaRa) and 0.5 µM of forward and reverse primers. For each gene analyzed, cDNA from 5×10^6^ bone marrow cells of CK-AML or NK-AML patients were used for amplification. Primers used were as follows:

MDM4FL-F: 5′-CAGCAGGTGCGCAAGGTGAA-3′

MDM4FL-R: 5′-CTGTG CGAGA GCGAG AGTCTG-3′

MDM4S-F: 5′-CAGCAGGTGCGCAAGGTGAA-3′

MDM4S-R: 5′-GCACTTTGCTGTAGTAGCAGTG-3

ABL-F: 5′-GAGTTCATGACCTAC GGGAACCT-3′

ABL-R: 5′-GGTACTCCATGGCTGACGAGAT-3′

PCR conditions: initial denaturation at 95°C 10 s; denaturation 95°C 15 s, annealing 60°C 30 s, 40 cycles. The average Ct for *MDM4FL*, *MDM4S*, and *ABL*, as well as the ΔCt (CtMDM4FL-CtABL or CtMDM4S-CtABL) was determined. NK-AML patients were set to 1 and relative expression graphed for *MDM4FL* and *MDM4S* mRNA in CK-AML patients. 2^−ΔΔCt^ was used for calculating relative quantification.

### Cell culture

HepG2 and 293T cell lines were obtained from the Institute of Cell Biology, Chinese Academy of Sciences, Shanghai, China. Cells were maintained in DMEM (Wuhan Boster, Biotechnology Ltd., Wuhan, China) supplemented with 10% fetal bovine serum (FBS; Gibco, Carlsbad, CA, USA), 100 U/ml penicillin, and 100 µg/ml streptomycin (Sigma, St. Louis, MO, USA). Nocodazole (Sigma) was dissolved in DMSO and used at either 0.1 µg/ml or 1 µg/ml.

### Lentivector infection

For construction of the pCDH1-MDM4FL-EF1-copGFP and pCDH1-MDM4S-EF1-copGFP, MDM4FL or MDM4S fragments and pCDH1-MCS1-EF1-copGFP plasmid were digested by *EcoR* I and *BamH* I respectively, and then linked with T4 DNA ligase (TakaRa). Plasmid sequences were confirmed by sequencing. Approximately 5×10^6^ 293T cells in 100 mm dishes was cotransfected with 10 µg pCDH1-MCS1-EF1-copGFP vector, pCDH1-MDM4FL-EF1-copGFP, or pCDH1-MDM4S-EF1-copGFP along with 10 µg packaging vector pPACKH1-GAG, pPACKH1-REV and pVSV-G using calcium phosphate precipitation. Media containing lentivirus were collected 48 and 72 hours after transfection and supernatant added to 5×10^5^ HepG2 cells/well of a 6-well plate with 8 µg/ml polybrene (Sigma). For infection, cells were centrifuged at 1400×*g* for 2.5 hours at 32°C. GFP-positive cells were screened by limiting dilution, expanded in culture, and GFP-positive cells were pooled. To confirm that MDM4 was transfected into HepG2 cells, the expression levels of MDM4FL and MDM4S proteins were evaluated by western-blot analysis.

### Cell cycle and cell proliferation assay

Cells stably expressing MDM4FL, MDM4S or vector control were cultured overnight and 0.1 µg/ml Nocodazole added the following day, and cells incubated for an additional 18 hours. Cells were stained by propidium iodine (PI) and cell cycle stage determined by flow cytometry (FCM). Cell proliferation was analyzed using the MTT assay. After 4 h incubation with MTT reagent, cells were lysed with DMSO for 10 min at 37°C and absorbance measured at 570 nm. The average percentage is shown for three independent HepG2 control, MDM4FL or MDM4S-expressing pools.

### Western-blot analysis

After treatment with 1 µg/ml Nocodazole for 18 hours, total protein was extracted from approximately 5–10×10^6^ control, MDM4FL or MDM4S-expressing cells, and stored at −80°C before use. Lysates (30 µg) were resolved by 8–12% SDS-PAGE and gels transferred to nitrocellulose membrane. Membranes were blocked with 5% nonfat milk in PBST for 1 h followed by primary antibody and incubation overnight at 4°C with gentle rotation. Membranes were washed twice with PBS containing 0.2% Tween 20 and incubated with appropriate secondary antibodies for 1 h at room temperature with gentle rotation. Membranes were then washed twice with PBST and incubated with Super Signal West pico (Pierce, Rockford, IL, U.S.A) for 1 minute and exposed to film. Images were captured using the Bio-Rad ChemiDoc Imager (Hercules, CA, USA). Data were normalized to GAPDH as a loading control. Primary antibodies used for detection were anti-P53 (1∶400, Boster), anti-P21 (1∶500, Bioworld Technology Inc, St. Louis, MO, USA), anti-BubR1 (1∶500, Bioworld Technology), anti-Securin (1∶500, Epitomics Burlingame, CA, USA), and anti-GAPDH (1∶500, Santa Cruz Biotechnology, Dallas, TX, USA). Secondary antibodies conjugated to HRP were used at 1∶2000 (Santa Cruz).

### Mitotic chromosome and karyotype analysis

Chromosomes spreads were prepared from control, MDM4FL, MDM4S- expressing cells, and stained with Giemsa. Images were acquired with Motic high quality scientific grade CCD cameras (Hong Kong). Metaphase cells (75 per sample) from control, MDM4FL and MDM4S expressing pools were scored for chromosomes. Three independent chromosome counts were obtained for each data set, and the rank sum test used to compare chromosome number dispersion. Kruskal-Wallis was used to compare the medians of three ranked variables. All statistical analyses were performed using SPSS 16.0 (IBM, Chicago, IL, USA) and *P*<0.05 was considered significant.

## Results

### CK-AML patients with wild-type *TP53* correlated with poorer prognosis than NK-AML patients

**Figure 1:**
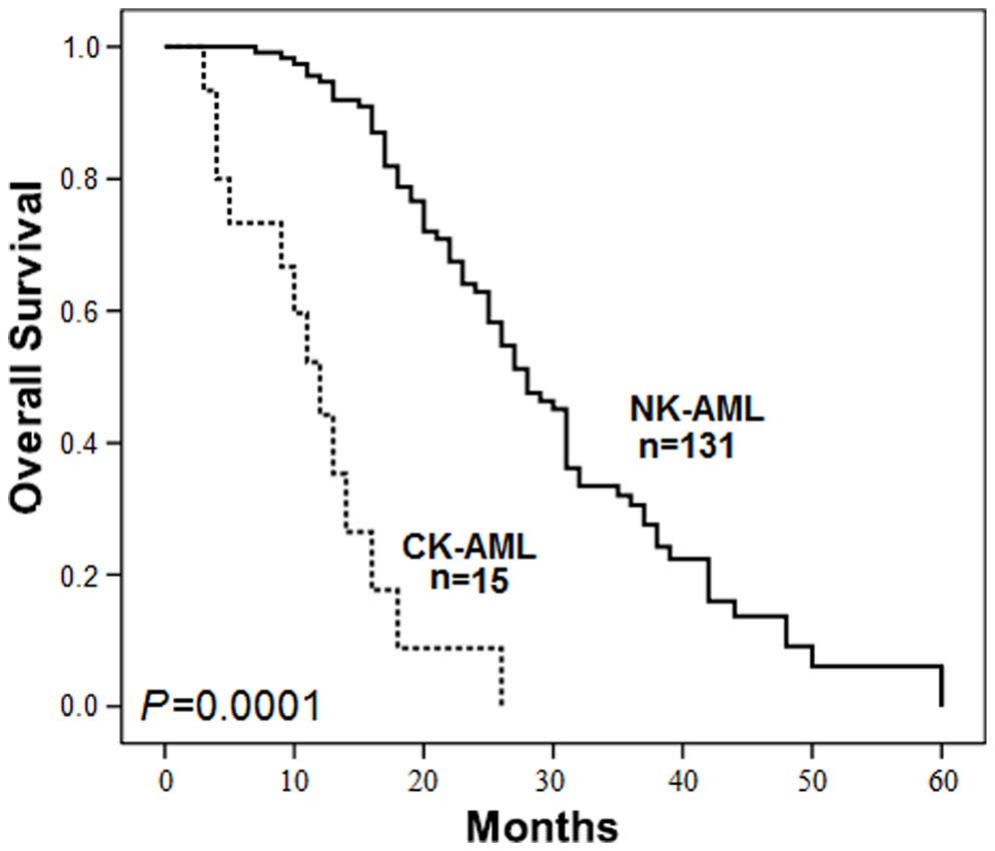
The overall survival of patients with NK-AML (solid line) and CK-AML (dotted line) analyzed by the Kaplan-Meier curve.

This study cohort included 15 CK-AML patients with wild-type *TP53*, a male/female ratio of 1.14 (8∶7) and median age of 59 years (range, 17–80 years), with seven patients (46.7%) ≥60 years. Two patients (13.3%) had WBC counts greater than 100×10^9^/L. One patient was classified with M0, four with M2, five with M4 and five with M5 according to FAB classifications. Karyotype analysis showed monosomy 5 (−5) (n = 2), and monosomy 7 (−7) (n = 4). Of the 15 patients monitored for therapy response and survival, four achieved complete response (CR) and two achieved partial response (PR). The median survival time was 292 days (range, 66–738 days). The clinical characteristics of the 15 CK-AML karyotypes were provided in Table 1. The overall survival (OS) of NK-AML patients was significantly higher than that of CK-AML patients (*P* = 0.001) (Figure 1).

**Table 1:** General information, peripheral white blood cell count, outcome, karyotype, and survival time of 15 AML patients with complex karyotype.

patient	Age	Sex	FAB classification	WBC^1^ (10^9^/L)	outcome	karyotype	Overall survival (Mon^6^)
1	59	F	M2	40.9	CR^2^	54, XXX.+8,+11,+15,+19,+20,+21,+22	3
2	76	M	M5	1.13	NT^3^	49, XY,+6, add(7)(p22),+8, add(11)(q25),+15	4
3	22	M	M2	18.9	CR	49, Y,+1,+5,−7,+8,−11, +13,+22,+t(11; 17) (q23; q21)	12
4	17	M	M5	106	PR^4^	51, XY,+13,+15,+16,+19,+22	17
5	44	M	M4	69.7	NR^5^	48, XXY,+1,−2,−5,+12, +18	9
6	62	F	M5	30	NR	50, XX,+1,−7,+8,+13,+15,+19	8
7	62	M	M5	0.77	NR	49, Y,+1,+5,−7,+8,+13, +21	11
8	64	F	M5	2.02	NR	50, XX, add(10)(p13),+16,+19,+21,+21	10
9	80	F	M4	83.7	NT	51, XX,−7,+13, +15,+21,+21,+21,+22	13
10	62	M	M4	26	PR	49, XY,+13,+19,+21	15
11	44	F	M4	79.9	NR	52, XXX,+10,+13,+16, −19,+21,+22,+der(19) t(11; 19)(q13; q21)	3
12	48	F	M0	4.17	NR	92, XXXX	2
13	55	M	M2	33.5	NR	47, XXY,+1,−2,−5,+19,+21,−22,+t(1; 17)(q31; q21)	25
14	67	M	M4	147	CR	56, XXXY,+1,−2,+10,+11,+12,+15,+20,+21,+21,+22	11
15	44	F	M2	2.48	CR	51, XXX,+8,+13,+15, +16	9

1 WBC were detected at the time of diagnosis;

2 CR: complete remission;

3 NT: no treatment;

4 PR: partial remission;

5 NR: no remission;

6 Mon: Month.

*TP53* mutation and deletions were detected by genome PCR sequencing and interphase FISH in 24 CK-AML cases, and 15 CK-AML cases were wild-type *TP53*. In order to rule out other genes mutation associated with poor prognosis of CK-AML, we detected RUNX1 mutations in 15 CK-AML patients and *FLT3-ITD* mutation in 131 *de novo* AML cases (15 patients with wild-type *TP53* and 116 NK-AML patients.). Among the 15 CK-AML patients, no *RUNX1* mutations were detected. The positive rates of *FLT3-ITD* in CK-AML and NK-AML were 20% and 23.2%, there was no significant difference between the two groups. (*P*>0.05).

### The relative expression levels of *MDM4S* and *MDM4FL* were higher in CK-AML than in NK-AML

**Figure 2:**
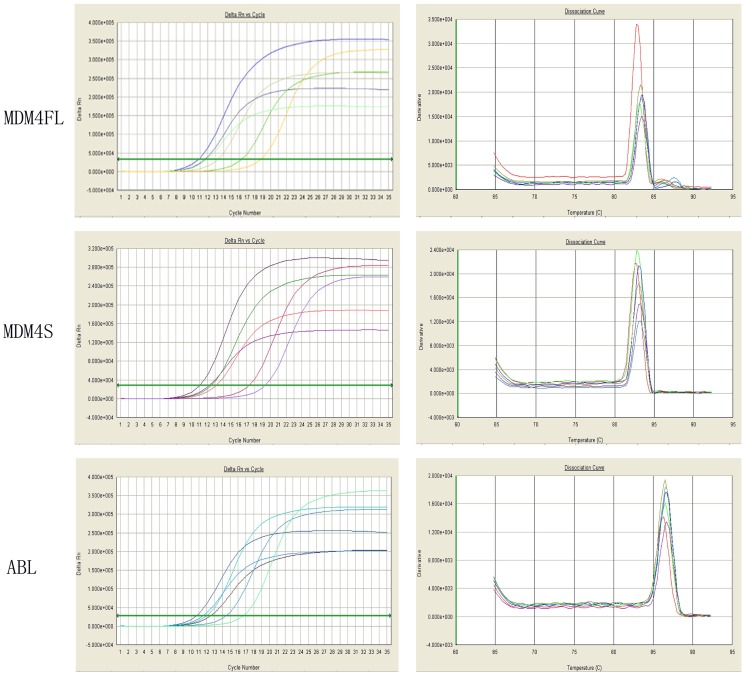
Amplification and melting curves of *MDM4FL*, *MDM4S* and *ABL.*

*MDM4FL* and *MDM4S* mRNA expression levels in CK-AML and NK-AML patients were assessed by real-time RT-PCR. The results indicated that normalized *MDM4FL* levels were 5.82 (1.67–20.28), while *MDM4S* levels were 22.25 (2.42–204.51). Both increased in CK-AML patients, with *MDM4S* showing a more notable increase (Table 2). The melting curve showed a single peak, suggesting a specific of amplified product (Fig. 2).

**Table 2:** The relative expression levels of *MDM4FL* and *MDM4S* mRNA.

GENE	ΔCt( 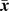 ±s) CK-AML	ΔCt( 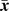 ±s) NK-AML	ΔΔCt	Normalized MDM4FL or MDM4S amount relative to NK-AML 2^−ΔΔCt^
*MDM4FL*	3.1300±2.5527	0.5882±1.2384	2.5418±1.8	5.82
*MDM4S*	6.1920±3.4192	1.7160±2.9743	4.4760±3.2	22.25

### The metaphase arrest was reduced and cell proliferation activity increased in MDM4-expressing cells

**Figure 3:**
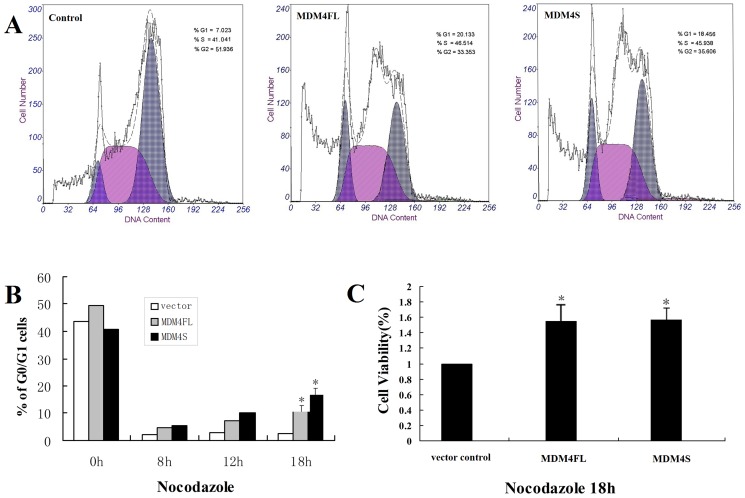
Cell cycle and cell proliferation analysis. A: DNA content detected by flow cytometry in MDM4FL, MDM4S-expressing or control cells. B: The proportion of G0/G1 phase cells at different time points after Nocodazole treatment. C: Cell proliferation assay. The average percentage of proliferating cells was increased in MDM4FL and MDM4S-expressing cells. *P<0.05.

HepG2 cells stably expressing MDM4FL, MDM4S or vector control were cultured overnight and 0.1 µg/ml Nocodazole added the following day and incubated for 18 hours. The percentage of M phase for control, MDM4FL and MDM4S-expressing cells were 51.94%, 33.35% and 35.61%, respectively. Compared with the control, there were fewer M phase cells in MDM4FL and MDM4S-expressing cells (*P*<0.05) (Fig.3A). We next examined the percentage of G0/G1 at different time points after Nocodazole treatment. Before Nocodazole treatment, the percentage of G0/G1 cells in all three lines was approximately 40–60%. Following Nocodazole treatment for 8 h, the percentage of G0/G1 cells in all three cell lines decreased sharply, and then gradually increased with prolonged treatment. At 18 h, the percentages of G0/G1 in MDM4FL and MDM4S-expressing cells were higher than that in control cells (*P*<0.05) (Fig. 3B). Finally, we examined cell proliferation activity using MTT assay after 18 hours of Nocodazole treatment. The proliferation activities were 0.807±0.071, 1.230±0.082 and 1.253±0.087 in control, MDM4FL, and MDM4S-expressing cells, respectively. Compared with the control, the average percentage of proliferating cells increased in MDM4FL and MDM4S-expressing cells (*P*<0.05) (Fig. 3C).

### p21 expression levels decreased in MDM4-expressing cells

**Figure 4:**
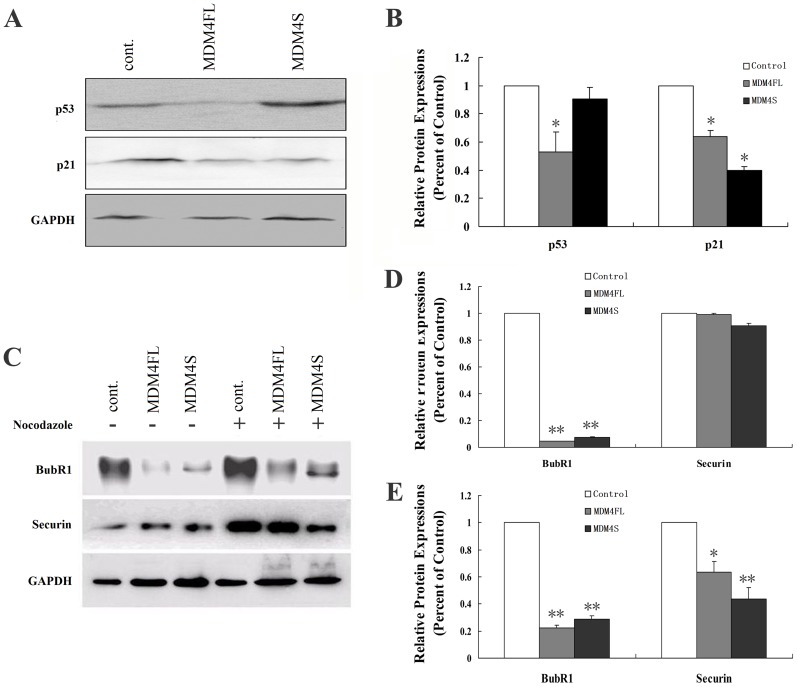
Dysregulation of p53 pathway and spindle checkpoint proteins are reduced in MDM4FL or MDM4S-expressing cells. A: Western blot analysis of p53 and p21 levels in control, MDM4FL, and MDM4S-expressing cells. B: Quantification of p53 and p21 expression levels in different cell groups. Levels were normalized against protein levels in control cells. * *P*<0.05. C: Western blot analysis of BubR1 and Securin in control, MDM4FL and MDM4S-expressing cells treated with 0.025% DMSO (−) or 1 µg/ml nocodazole (+) for 18 hours. D: BubR1 and Securin expression levels following treatment with 0.025% DMSO. E: BubR1 and Securin expression levels following treatment with 1 µg/ml Nocodazole. * *P*<0.05, ** *P*<0.01. Immunoblot for GAPDH confirms relative protein loading.

To explore whether MDM4 overexpression inhibited the activity of P53 pathway, p53 and p21 expression levels were examined in the overexpressed MDM4 cell model. Our data showed that compared with control, p53 expression level decreased in MDM4FL-expressing cells (*P*<0.05), but it did not decline significantly in MDM4S-expressing cells (*P*>0.05). However, the p21 expression levels decreased in both MDM4FL and MDM4S-expressing cells compared with control (*P*<0.01) (Fig.4A–B).

### BubR1 and Securin expression levels decreased in MDM4-expressing cells

The spindle checkpoint proteins, BubR1 and Securin, were assessed by western blot in control, MDM4FL or MDM4S-expressing cells. The results showed that the expression levels of BubR1 and Securin in MDM4FL and MDM4S-expressing cells decreased following Nocodazole treatment. However, control cells exhibited increased Securin levels, consistent with previous reports [14] that APC activity is required to destabilize Securin (Fig.4C–E).

### Polyploidy and aneuploidy in MDM4FL and MDM4S-expressing cells

**Figure 5:**
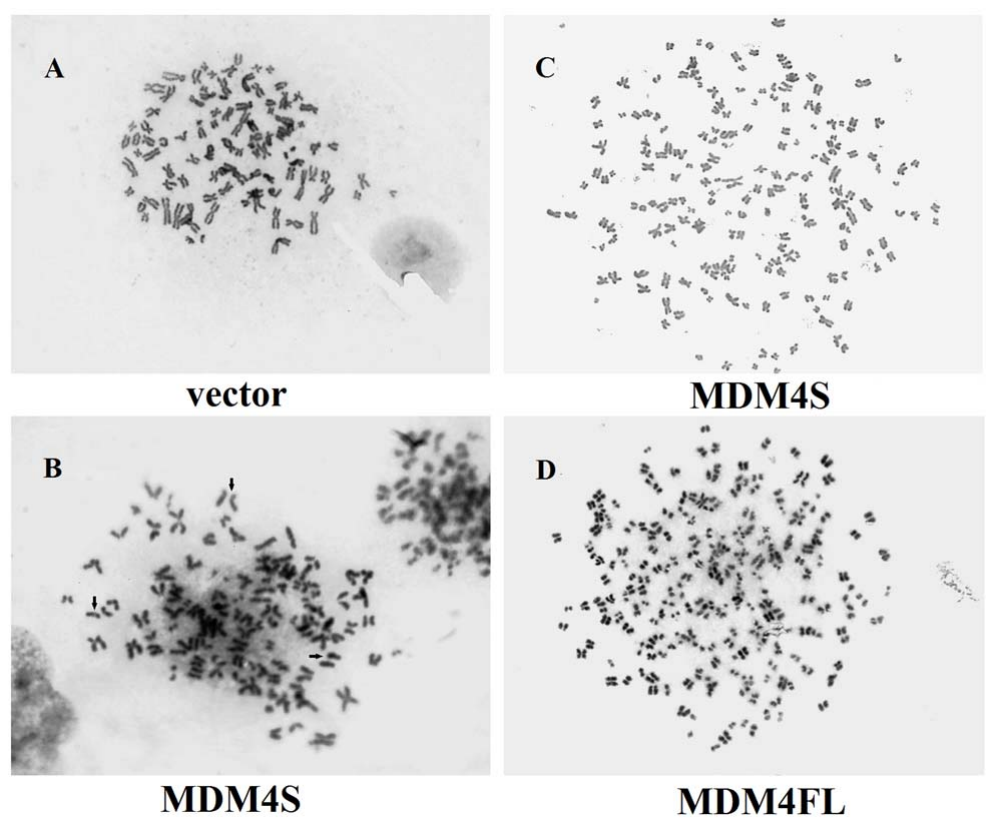
Prometaphase and mitotic of MDM4FL and MDM4S-expressing cells. A: Chromosome spread of a prometaphase vector control cell. B: Premature sister chromatid separation in an MDM4S prometaphase cell (indicated by arrows). C: Polyploidy in a MDM4S cell. D: Endoreduplication of a MDM4FL cell.

**Figure 6:**
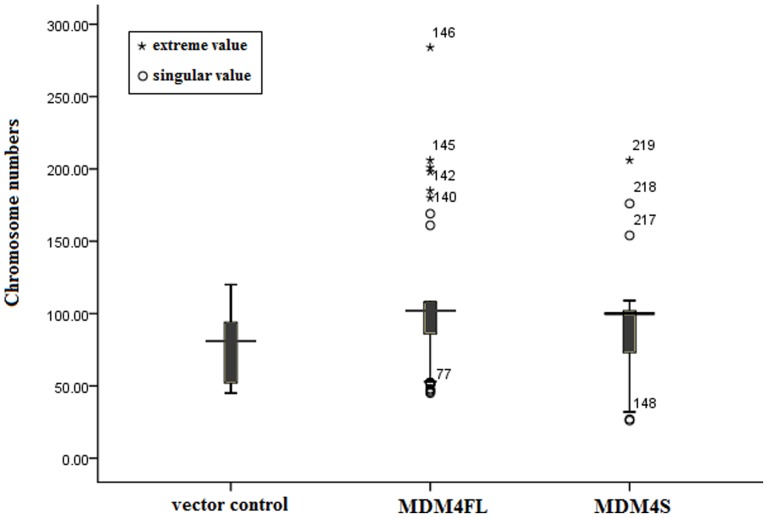
Boxplot reflecting the central tendency and dispersion tendency of the chromosome numbers of each group. * singular values, ^o^extreme values.

We then monitored chromosome number, premature sister chromatid separation and polyploidy or endoreduplication in control, MDM4FL and MDM4S-expressing cells. Karyotype analysis showed that prematurely dissociated sister chromatids prior to anaphase, polyploidy or endoreduplication were observed in MDM4FL or MDM4S-expressing cells, but not in control cells. (Fig. 5). Chromosome number data are expressed as medians (25th and 75th percentile). The median chromosome numbers were 81(52, 94) (range 45–120), 102 (86, 108) (range 45–284), and 100 (73, 102) (range 26–206) for control, MDM4FL and MDM4S-expressing cells, respectively (Kruskal-Wallis evaluation, *P*<0.05). Therefore, we conclude that at least one of these chromosome numbers had a different ranking distribution relative to the others. Boxplot analysis suggests that the MDM4S and MDM4FL cells most likely have different distributions from control cells. The chromosome numbers of each group reflects the range of chromosome numbers for MDM4S and MDM4FL, which were much more diverse. There were several singular and outlier values in MDM4FL or MDM4S-expressing cells, however they were not found in control cells (Fig. 6).

## Discussion

About 70% of CK-AML cases contain p53 mutations, and are often associated with poor prognosis [4], [5]. Cell cycle regulation is closely related to the transcriptional activation of p53. Several studies have shown that Nocodazole, a spindle inhibitor, when applied to p53−/− mouse fibroblasts, become polyploidy because of endoreduplication. This suggests an important role for p53 in regulating spindle checkpoint in mice [15]–[17]. p53 dysfunction leads to decreased p21 expression and a weakened spindle checkpoint. A cell with a chromosome aberration and with a weakened spindle checkpoint will continue to proliferate and exhibit aneuploidy or complex karyotype [18]. In this study, we ruled out mutations of some genes related to poor prognosis of CK-AML, including *RUNX1* [19], and *FLT3-ITD* in 15 CK-AML patients lacking *TP53* mutation. These results implied that there might be other important molecular events involved in the leukemogenic mechanisms that occur in CK-AML patients with wild-type *TP53*.

MDM4 is a negative regulation factor of p53, which exerts its effect by binding p53. *MDM4* has several transcript variants [20], with the *MDM4S* transcript obtained by exon 6 deletion, resulting in a truncated protein containing only the p53 binding domain. It has been reported that MDM4S affinity to p53 is approximately 10-fold higher than that of MDM4FL [21]. High levels of *MDM4S* mRNA expression are associated with short treatment free survival [11] and its overexpression was significantly correlated with an unfavorable prognosis in soft-tissue sarcoma patients [10], [22]. Our results showed that *MDM4FL* and *MDM4S* expression levels were elevated in CK-AML patients relative to NK-AML patients. We thus speculate that *MDM4* overexpression may be involved in the leukemogenic mechanisms of CK-AML patients with wild-type *TP53*.

To prove the above speculation, we tried to find a leukemic cell line with wild-type p53 in the catalog of the American type culture collection (ATCC). However, all myeloid cell lines either contain mutant p53 or do not express p53 [23]–[26]. Taking into account the purpose of our experiments is just to investigate if MDM4 overexpression would influence p53 signal pathway in cancer cell with normal p53, we decided to choose other appropriate cancer cell to continue the study. The HepG2 cell line expresses wild-type p53, normal levels of MDM4, and low levels of MDM4S [27]. These characteristics were appropriate for our experiments. MDM4-expressing HepG2 cells displayed a reduced mitotic index following Nocodazole treatment, suggesting a failure in a subset of cells to undergo mitotic arrest through a functional spindle checkpoint. Additionally, MDM4-expressing cells had reduced levels of p21, an important effector molecule downstream of p53. This indicates that overexpression of MDM4FL or MDM4S inhibits p53 signaling pathway.

BubR1 is a critical component of the spindle checkpoint. BubR1 performs several roles during mitosis and ensures accurate chromosome separation [28]. Securin is one of the main substrates of APC/C [29]. The expression levels of BubR1 and Securin decreased in MDM4-expressing cells following Nocodazole treatment, suggesting that APC may be active in these cells because of a spindle checkpoint decline. However, following Nocodazole treatment, control cells had increased levels of Securin. These results indicate proper functioning of the spindle checkpoint and an inactive APC in control cells. Cells that continue to proliferate with an attenuated spindle checkpoint should missegregate chromosomes and become aneuploid. Previous reports indicate that Securin loss can lead to karyotype changes in cell lines [30]. Therefore, it is possible that the spindle checkpoint and APC activity, through BubR1 and Securin down-regulation, contribute to the attenuation of cell cycle checkpoints.

Suppression BubR1 results in a dysfunction spindle checkpoint and leads to abnormal mitosis and aneuploidy [31]. CK-AML patients have been defined as the presence of at least five clonal aberrations or at least three abnormalities in the absence of t(8; 21), inv(16)/t(16; 16), and t(15; 17) [32]. Complex karyotype, like aneuploidy, may result from chromosome missegregation during mitosis. Our results suggest that MDM4 overexpression may cause aneuploid or polyploidy. We have not observed the association between specific chromosomal abnormalities and MDM4 overexpression because we only have 15 CK-AML patients with wild-type *TP53*. Although it is not well known if there is a causal relationship between MDM4 overexpression and aneuploidy, these date raise the possibility that MDM4 overexpression plays a role in CK-AML pathogenesis. It will be necessary to evaluate more patients and to further explore the molecular mechanisms of MDM4 overexpression and to develop targeted therapies for CK-AML patients. At least in theory, restoration of p53 function is a potential therapeutic approach in leukemia. Bista M et al [33] reported that SJ-172550, an inhibitor of the interaction between MDM4 and p53, may be a new option for the treatment of CK-AML. Their results suggest that the combination of a MDM4 inhibitor and traditional chemotherapy for refractory CK-AML may be worth evaluating.

*MDM4* expression levels were elevated in CK-AML patients relative to NK-AML patients, MDM4-overexpressing HepG2 cell lines had a reduced mitotic index, reduced p21, BubR1 or Securin expression levels following Nocodazole treatment, and MDM4-overexpressing cells were aneuploidy or polyploidy. Based on data presented in this study, we speculate that the leukemogenic mechanism of CK-AML without *TP53* alternations is partly due to the p53 signaling pathway inhibited and the spindle checkpoint weakened by MDM4 overexpression. MDM4 may be a novel therapeutic target in the treatment of CK-AML patients with wild-type *TP53*.
